# The impact of integrating medical assistants and community health workers on diabetes care management in community health centers

**DOI:** 10.1186/s12913-018-3710-9

**Published:** 2018-11-20

**Authors:** Hector P. Rodriguez, Mark W. Friedberg, Arturo Vargas-Bustamante, Xiao Chen, Ana E. Martinez, Dylan H. Roby

**Affiliations:** 1 Division of Health Policy and Management, School of Public Health, University of California, Berkeley, 50 University Hall, Room 245, Berkeley, CA 94720 USA; 2 RAND Corporation, Boston, MA USA; 3 Department of Health Policy and Management, Fielding School of Public Health, University of California, California, Los Angeles USA; 4 UCLA Center for Health Policy Research, Fielding School of Public Health, University of California, California, Los Angeles USA; 5 Division of Health Services Management, School of Public Health, University of Maryland, College Park, MD USA

**Keywords:** Community health centers, Medical assistants, Community health workers, Diabetes care management, Patients’ experiences, Implementation research

## Abstract

**Objective:**

To compare the impact of implementing team-based diabetes care management involving community health workers (CHWs) vs. medical assistants (MA) in community health centers (CHCs) on diabetes care processes, intermediate outcomes, and patients’ experiences of chronic care.

**Data sources:**

Clinical and administrative data (*n* = 6111) and patient surveys (*n* = 698) pre-intervention and post-intervention. Surveys (*n* = 285) and key informant interviews (*n* = 48) of CHC staff assessed barriers and facilitators of implementation.

**Study design:**

A three-arm cluster-randomized trial of CHC sites integrating MAs (*n* = 3) or CHWs (*n* = 3) for diabetes care management compared control CHC sites (*n* = 10). Difference-in-difference multivariate regression with exact matching of patients estimated intervention effects.

**Principal findings:**

Patients in the CHW intervention arm had improved annual glycated hemoglobin testing (18.5%, *p* < 0.001), while patients in the MA intervention arm had improved low-density lipoprotein cholesterol control (8.4%, *p* < 0.05) and reported better chronic care experiences over time (*β*=7.5, *p* < 0.001). Except for chronic care experiences (*p* < 0.05) for patients in the MA intervention group, difference-in-difference estimates were not statistically significant because control group patients also improved over time. Some diabetes care processes improved significantly more for control group patients than intervention group patients. Key informant interviews revealed that immediate patient care issues sometimes crowded out diabetes care management activities, especially for MAs.

**Conclusions:**

Diabetes care improved in CHCs integrating CHWs and MAs onto primary care teams, but the improvements were no different than improvements observed among matched control group patients. Greater improvement using CHW and MA team-based approaches may be possible if practice leaders minimize use of these personnel to cover shortages that often arise in busy primary care practices.

## Background

The effectiveness of team-based models of primary care that include registered nurse care management [1] and pharmacist-led medication management [2] in improving diabetes care quality and patient self-management is well established. Despite evidence of effectiveness, these models for managing diabetes care are not financially feasible for many community health centers (CHCs) that serve low-income patients [3]. Team-based models that involve patient panel management by medical assistants (MAs) and/or community-based care management by community health outreach workers (CHWs) are relatively more financially feasible for CHCs to adopt and implement. Despite the large disease burden of diabetes among vulnerable populations [4, 5], the effect of these lower cost and scalable team-based approaches on improving the diabetes care management remains unclear.

Previous research has demonstrated the patient health benefits of diabetes education, especially programs and interventions that emphasize patient self-management for vulnerable patients [6, 7], and MAs and CHWs can provide self-management support to diabetic patients and change patient knowledge and behavior and, in some instances, health outcomes [8–10].

MAs are projected to be among the fastest growing occupational groups between 2016 and 2026 [11] and are the fastest growing group on adult primary care teams [12]^,^. Demand for MA support and services in health coaching and panel management roles is likely to increase as a result of changing external incentives toward value-based payment [13]. The potential positive impact of MA health coaching and panel management interventions on patient outcomes and experiences of care in underserved settings highlights the promise for expanding MA responsibilities to support diabetes care management [14–17].

CHWs also have competencies that enable them to support patients with diabetes. They are peer educators whose goal is to promote health in their community through information distribution, assistance, social support, and organizing community networks [18, 19]. CHW roles have included: member of care delivery teams, navigator, screening and health education provider, outreach and enrolling agent, and organizer [9]. CHWs generally work with patients in community settings and some conduct home visits or support groups. CHWs can improve diabetes self-management skills among patients [20]. However, few studies have assessed the effectiveness of CHWs in improving outcomes of care in routine settings [21, 22], and while several patient-level randomized controlled trials have been conducted [23], we know of no studies that have examined the effectiveness of routine implementation of CHWs in CHCs using a cluster randomized research design.

We compared the impact of implementing CHWs and MA with expanded roles and responsibilities for managing diabetes care in CHCs that care for predominantly low-income Latino Spanish-speaking or Chinese patients. To our knowledge, no research has compared the impact of integrating MAs vs. CHWs as members of CHC primary care teams compared to a control group. We hypothesized that MA panel management and CHW integration would have different impacts on diabetes care management. Because MAs are office-based and focus on ensuring that patients receive appropriate treatments and tests [13], we hypothesized that team-based models of diabetes care management involving MAs would improve process of care measures to a greater degree than teams with CHWs. In contrast, CHWs’ roles focus on patient activation and self-management in community settings [9], where individuals make important diet and lifestyle choices. As a result, their contributions to diabetes management may be more likely to result in patient engagement and self-management compared to MAs. Given the challenge of measurably improving patients’ experiences [24], we anticipated that changes in patients’ experiences of care resulting from implementing the team-based models would be modest and similar for sites integrating CHW and MAs.

## Methods

### Study design

A cluster-randomized trial using effectiveness-implementation Type II study design [25] was conducted with the simultaneous goals of examining the impact of integrating MAs or CHWs into care teams on patient outcomes and assessing barriers and facilitators of routine integration of new care team members, roles, and responsibilities. We recruited 16 CHC sites located in three counties in Northern California that were all affiliated with the same regional community clinic association. The participating CHCs served primarily low-income, Latino and Chinese patients and patients. All CHC sites studied have bilingual and bicultural staff that can provide care in patients’ language preference.

Prior to site randomization from June–August 2011, we conducted practice surveys of each site director (*n* = 15, response rate: 94%) and 281 adult primary care clinicians and staff members from each participating CHC (response rate: 81%) to assess baseline practice climate and organizational factors that would impact the effectiveness of clinic efforts to implement team-based models of care. The site director survey assessed the diabetes care management capabilities of CHC sites, including use of registries, self-management education, use of data for improvement purposes, and connection to community resources [26]. A validated survey of CHC adult primary care clinicians and staff was used to assess teamwork and practice climate [27].

Using the response of the site director and mean responses to the clinician and staff surveys, we dichotomized practices into “high” (top eight) vs. “low” (bottom eight) on each of the following composite measures: 1) diabetes structural capabilities, 2) primary care team functioning, 3) practice size (mean number of clinicians/staff: 18). Using cluster analyses, we grouped practices into three sampling strata based on the most common combinations of these three composite measures and randomly assigned practices within each strata to the MA intervention (*n* = 3), CHW (*n* = 3), and control (*n* = 10) group arms of the cluster-randomized trial. Stipends equivalent to the costs of personnel and benefits of new MA and CHW team members were provided to each CHC intervention site for one year (2012). Each participating site received a stipend each year (2011–2013) to defray the costs of clinic and administrative data collection and reporting required of research study participation.

### Interventions

In most cases (5 of 6), existing MAs were promoted to take on the new responsibilities for diabetes care management. All CHCs hired replacements for the MAs taking on the project roles. MA and CHW personnel from intervention CHC sites were trained on health coaching and panel management for diabetes care by expert trainers prior to the intervention, between October and December 2011, over three in-person six-hour sessions. Table 1 summarizes the roles and responsibilities for managing diabetes care emphasized in MA vs. CHW training and implementation. All intervention personnel received training in motivational interviewing. The MA training emphasized office-based panel management activities for diabetic patients, including maintaining the diabetes registry, reviewing the panel at regular intervals, following up on primary care clinician instructions, assisting with medication reconciliation, and targeting patients for appropriate interventions and referral to community resources. The MAs also received a brief module on health coaching strategies. In contrast, the CHW training emphasized a broader range of patient health coaching skills, including helping patients set their agendas for clinicians visits, making sure patients understand what their clinicians want them to do, determine whether patients agreed with their care plans, provide support to patients’ efforts in adopting healthy behaviors, assist patients to improve medication understanding and adherence [28]. The CHWs also received a brief, less extensive module on panel management activities. Once trained, the personnel followed role-specific diabetes care management protocols developed by the participating clinics’ quality improvement teams in collaboration with the regional community clinic association.

**Table 1:** A comparison of medical assistant and community health worker roles in managing diabetes care for training and implementation

	Medical assistant intervention arm	Community health worker intervention arm	Control group arm^a^
Pre-visit			
Discuss the patient case with the physician	X		
Agenda setting with the patient	X	X	
Ordering routine services	X	X	X
History tracking	X		
During the Visit			
Document physician findings	X		
Send electronic prescriptions to pharmacy	X		
Write prescriptions for the physician to sign	X		
Post-visit			
Discuss patients’ concerns	X	X	
Recapitulate the advice given by the physician	X	X	
Set goals with the patient	X	X	
Make sure that patients can navigate the system		X	
Between Visits			
Provide culturally appropriate and accessible health education and information	X	X	
Assure that people with diabetes receive the services they need	X	X	X
Follow up over the phone	X	X	
Offer informal counseling and social support		X	
Provide information to families to support the lifestyle changes of patients with diabetes		X	
Build individual and community capacity		X	

^a^Community health center sites in the control group did not implement major changes to diabetes care processes during the study period, as verified by key informant interviews during the study period. Most control CHC sites, however, had some support for pre-visit ordering of routine diabetes services and follow up to assure services were received for patients in general (not specific to patients with diabetes)

Intervention CHC site administrators and frontline personnel received ongoing technical assistance from a regional community clinic association, including managing and reporting clinical and administrative data central to assessing diabetes care processes and intermediate outcomes, reinforcing motivational interviewing skills among MA and CHW staff, and didactic refresher trainings on evidence-based diabetes care management. The intervention period was from January–December 2012, but all practices continued to use the team-based models through 2013.

### Clinical and administrative data

The patient analytic sample was defined as diabetic using ICD-9 codes 250, 357.2, 362.0, 366.41, and 648.0. To supplement missing information in encounter data, we used additional methods to identify patients with diabetes, including pharmacy, billing/claims, and other encounter record data. We analyzed patient-level information on demographic information, CHC visits, and clinical measures of blood pressure, low-density lipoprotein cholesterol (LDL-C), and glycated hemoglobin (HbA1c). Healthcare Effectiveness Data Information Set (HEDIS) comprehensive diabetes care measure definitions were used to construct dichotomous outcome measures of control: HbA1c <8.0%, blood pressure <140/90 mmHg, and LDL-C<100 were considered “controlled”.

### Patient experience surveys

The early intervention patient experience survey of adult diabetics was fielded from July–August 2012 and the post-intervention survey of baseline respondents was fielded from July–August 2013. Surveys asked about care experiences during the prior six months. A total of 167 randomly sampled patients per control practice and up to 400 patients at each of the intervention practices were sampled for the survey. Sampled patients had at least 2 visits with a diabetes diagnosis during the pre-intervention year (2011). The survey was administered in English, Spanish or Chinese and included a $10 gift card. Patients were sent a survey in the mail with the gift card, and non-respondents received a second survey and then were called by phone with up to 8 attempts for each patient to complete the survey with an interviewer. Patients contacted by phone were consented by phone, while patients returning the survey consented by returning the survey. The pre-intervention (*n* = 1298; RR = 47%) and post-intervention (*n* = 698; RR = 65%) survey responses were merged to generate the cohort for the survey analyses.

Survey questions included the Clinician & Group - Consumer Assessment of Healthcare Providers and System (CG-CAHPS) survey [29], Patient Assessment of Chronic Illness Care (PACIC-11) measure [30], the Problem Areas in Diabetes (PAID-5) scale [31], and a count of self-reported hypoglycemic events over the past month. [32] Like many CHCs nationally, the sampled CHCs did not empanel patients to specific primary care clinicians (PCCs) due to high PCC turnover [33] and instead assigned patients to teams or to the entire practice. Accordingly, we adapted the standard CG-CAHPS questions to reference “doctors and nurses at this clinic” rather than “your personal doctor”. PACIC-11 is widely used to evaluate the delivery of chronic care management activities for a variety of conditions, including diabetes [30, 34]. PAID-5 is a psychometrically robust short-form measure of diabetes-related emotional distress [31]. Finally, a hypoglycemia measure was included because it was an important adverse event and contributor to poorer diabetes outcomes [32]. We used the half-scale rule [35] to calculate composites scores, whereby respondents had to complete at least half of the items comprising the composite measure for a score to be calculated. For ease of interpretation, we transformed the unweighted average of the survey composite questions to a 0 to 100 scale when calculating composite scores.

### Key informant interviews

Mid-intervention semi-structured interviews of CHC clinician and staff key informants were conducted from May–August 2012 and post-intervention interviews of the same key informants were conducted between March–June 2013. A total of 48 interviews were conducted with 28 individual clinicians and staff who provided consent to participate. This approach allowed for in-depth data collection to identify changes in diabetes care processes, and assess facilitators and barriers of implementation. Although the control sites were not encouraged to implement any changes, we conducted interviews to understand any changes to diabetes care processes and capabilities to shed light on any over-time changes in diabetes care processes among intervention clinics. All interviews were conducted by telephone or in-person using an interview guide and were audio recorded and transcribed. The aim of the interviews was to learn about changes in diabetes care at the CHC site, and perceived barriers and facilitators of implementing the team-based approach involving MAs and/or CHWs [36]. Informed consent for recording and transcribing the interviews was obtained for all participants at the beginning of each interview. Interview participants received a $25 gift card for participating in each interview. The human research ethics committee at University of California Los Angeles approved the study protocol (IRB#10–000596).

### Analytic samples

Data collection and reporting was complicated by the diverse clinical information and administrative systems being queried at each of the sites to supply the patient-level data. CHCs have high proportions of patients with unstable home and work environments that impact their ability to follow-up and manage their diabetes. In our CHC patient population, a total of 11,249 unique adult diabetic patients had at least one visit with a diabetes diagnosis at one of the 14 CHC sites in 2011. Since we used the SUPREME-DM definition [37] to define our analytic sample, we included patients with 2 or more visits, excluding 4163 (37.0%) patients who had only one visit. Of the remaining 7068 diabetic patients with 2 or more visits during 2011, 975 patients (13.8%) did not have a documentation of each outcome measure (HbA1c, LDL, and blood pressure) during the 2-year period (2011–2012) and were excluded from the analyses. The final analytic sample is 6111 adult diabetic patients.

The clinic randomization process attempted to balance diabetes structural capabilities, primary care team functioning, and practice size between the two intervention arms and control arm, and did not consider the underlying patient profile [38] of the participating practices given that the modest CHC clinic sample size precluded use of more than three factors. To address the potential bias of unbalanced data on estimating intervention effects and modest patient sample size that limited direct comparisons of these groups, separate control groups were constructed for the MA and CHW intervention groups using exact matching on a set of covariates. The exact matching algorithm matched patients on age (10-year bands), sex, race/ethnicity, primary language, and health insurance status (uninsured vs. insured). Matching resulted in at least one match (average of 3.6) per intervention patient. This yielded more valid comparisons between each intervention group and a matched control group for DID regression analyses.

### Statistical analyses

First, we performed a parallel comparison of sociodemographic and health characteristics for each intervention group (MA and CHW) and the matched control group. For these unadjusted analyses, we used t-tests to examine differences in continuous measures and **χ**^2^ tests to examine differences in categorical variables by race/ethnicity and language and compared changes in indicators between intervention and control arms over time. These tests used robust standard errors to handle patient clustering within CHC sites.

A difference-in-difference (DID) regression approach was used to assess the over-time impact of the CHW and MA interventions on diabetes care management compared to a matched control group matched on a set of covariates. Multilevel regression models were used to account for the clustering of patients within CHC sites using site random effects. We compared the binary specifications of HEDIS intermediate outcome measures of LDL-C, HbA1c, and blood pressure. These DID regression models controlled for age, sex, education, self-rated health and insurance status. Due to the collinearity of intervention assignment and racial and ethnic composition of patients at the CHC sites, the multivariate regression models did not include race/ethnicity as a covariate. To assess the robustness of our estimated intervention effects on diabetes processes and intermediate outcomes, we examine the impact of including patients with incomplete outcome data on diabetes care processes and outcomes as a sensitivity analysis.

A similar regression approach was used for the patient-reported experience and symptom outcome measures. Instead of matching, we included more control variables (patient reported use of insulin, self-rated health) because we had survey and administrative data for this smaller random subgroup of patients. Using the final regression models, we estimated the adjusted estimates of intervention effects for each of the outcome measures (i.e., CG-CAHPS, PACIC-11, hypoglycemia, HbA1c, LDL-C, and blood pressure). Significance was determined using a 2-tailed alpha of 0.05. All analyses were conducted using Stata 14.0.

### Interview data analyses

Transcribed interviews were analyzed using Atlas.ti. We used a qualitative content analysis with a directed approach. [39] A framework of codes was developed by two researchers, and checked for consistency. A log was maintained of emerging codes and used for adapting the coded framework. The adapted framework was reviewed for consistency by two researchers not involved in the coding process. The final list of codes was applied to all interviews by two investigators for each of the key informant roles. Coding discrepancies were reconciled during regular research team meetings. Output stratified by intervention group was analyzed and themes were summarized and compared across groups.

## Results

Of the 16 CHC sites that were initially randomized into the intervention and control groups, 2 sites (1 from the control group and 1 from the MA intervention group) dropped out of the research study during the early intervention period (January–February 2012) because of data reporting challenges that prevented them from fully participating.

### Sample characteristics

Patients in the MA study arms were older and more likely to be uninsured and of Asian descent compared to CHW arm patients. Patients of the CHW study arms were more likely to be of Latino descent, had higher BMI levels, and were more likely to have diagnosed and documented mental health co-morbidities, e.g., depression and anxiety, than MA intervention arm patients. The exact matching method balanced patient age, race/ethnicity, between each intervention group and its separately constructed control group (Table 2).

**Table 2:** Adult diabetic patient characteristics for the overall and analytic samples, 2011–2013

Patient characteristic (% or Mean and SD)	Overall sample	Community Health Worker (CHW) Arm	CHW control group arm	Medical Assistant (MA) Arm	MA control group arm
n	6111	686	2466	644	2315
Male (%)	41.9	30.3	30.3	47.8	47.8
Age (%)					
26–35	3.0	8.5	8.5	2.2	2.2
36–45	9.1	16.2	16.2	7.3	7.3
46–55	20.5	26.5	26.5	22.4	22.4
56–65	32.2	24.9	24.9	38.2	38.2
65–75	20.4	17.9	17.9	19.9	19.9
75+ (reference)	14.9	6.0	6.0	10.1	10.1
Race/Ethnicity/Language (%)					
Asian- Asian language speaking	49.8	1.2	1.2	72.8	72.8
Asian- English speaking	6.0	7.3	7.3	11.2	11.2
Latino- English speaking	8.2	20.1	20.1	3.0	3.0
Other English speaking (reference)	7.3	9.8	9.8	4.2	4.2
Latino- Spanish speaking	28.7	61.7	61.7	8.9	8.9
Uninsured (%)	31.9	16.0	16.0	53.3	53.3
Body-Mass Index (BMI) (%)					
BMI (Normal, BMI ≤25) (reference)	27.6	12.5	13.4	39.0	39.1
BMI (Overweight, BMI ≥26 and ≤ 30)	32.9	31.3	39.4	16.3	16.1
BMI (Obese I & II, BMI ≥31 and ≤ 40)	5.8	8.8	11.3	2.6	2.8
BMI (Obese III, BMI ≥41))	24.9	29.9	26.3	37.0	35.3
Missing BMI	8.8	17.5	9.6	5.1	6.7
Co-Morbidities					
Cardiovascular Disease (%)	8.2	5.7	6.8	6.2	7.4
Respiratory Problems (%)	7.7	8.9	8.6	4.0	5.3
Mental Health (%)	13.9	22.2	20.0	7.1	8.5
Number of CHC Visits During the Study Period (%)					
<=3	8.2	6.2	9.1	7.3	10.3
4–8	39	37.6	34.9	51.9	45.8
9–11	24.5	26.3	24.3	24.1	24.4
12+	28.3	32.9	31.7	16.8	19.5

### Clinical process and intermediate outcome results

In adjusted DID analyses, patients in the CHW intervention arm had improved annual glycated hemoglobin testing (18.5% points, *p* < 0.001), but the differences were no different than improvements in HbA1c testing among patients in the CHW control group (Table 3). LDL-C testing, however, improved significantly more over time for the CHW control group compared to the CHW intervention group (9.8% points vs. 0%, *p* < 0.01).

**Table 3:** Comparison of community health worker vs. control group arm on overtime changes on diabetes processes of care and intermediate outcomes of care

	Community Health Worker (CHW) Arm (*n* = 686)			CHW Control Group Arm (*n* = 2466)			
	Pre	Post	*∆* Over Time	Pre	Post	*∆* Over Time	Difference-in-Difference
Glycated hemoglobin (HbA1c) tested (%, 95 CI)	63.9 (43.4, 84.4)	82.4 (69.3, 95.6)	18.5 (9.5, 27.6)***	71.9 (61.5, 82.2)	85.5 (78.9, 92.0)	13.6 (9.1, 18.2)***	4.9 (−5.2, 15.0**)**
HbA1c controlled below 8.0% (%, 95 CI)	87.3 (83.3, 91.4)	87.9 (83.9, 91.8)	0.6 (−3.6, 4.6)	88.2 (86.0, 90.5)	86.9 (84.6, 89.2)	−1.3 (− 3.3, 0.6)	1.8 (− 2.7, 6.3)
Low density lipoprotein cholesterol (LDL-C) tested (%, 95 CI)	69.2 (53.8, 84.6)	69.2 (53.8, 84.6)	0.0 (− 5.8, 5.7)	67.7 (58.8, 76.7)	77.5 (70.2, 84.8)	9.8 (6.5, 13.0)***	−9.8 (− 16.4, − 3.2)**
LDL-C- controlled below 100 mg/dL (%, 95 CI)	66.0 (58.4, 73.7)	65.8 (57.7, 74.0)	−0.2 (−7.2, 6.8)	60.9 (56.7, 65.1)	63.0 (58.8, 67.2)	2.1 (− 1.3, 5.6)	−2.3 (− 10.2, 5.5)
Blood Pressure- controlled below 140/90 mmHg (%, 95 CI)	57.6 (41.6, 73.7)	63.7 (48.7, 78.7)	6.0 (− 1.5, 13.6)	63.1 (54.8, 71.4)	62.8 (54.4, 71.1)	−0.3 (− 3.2, 2.4)	6.4 (− 1.6, 14.5)

Note: Control variables used for adjustment: patient age, sex, and insurance status. Bold denotes statistically significant (*p* < 0.05) differences between the intervention and control group. Statistically significant differences are denoted in the following manner: * *p* < 0.05, ** *p* < 0.01, ***p < 0.001

The MA intervention group patients improved LDL-C control (8.4% points, *p* < 0.05) over time, but the improvements were no different than patients in the MA control group (Table 4). HbA1c testing, however, improved more for the MA control group compared to the MA intervention group (5.8% points vs. 1.0% points, *p* < 0.05). There were no other differential changes over time for the other diabetes care processes or intermediate outcomes for the MA intervention and control group arms. The final regression results were generally consistent in sensitivity analyses that included patients with one or more missing test values during the study period (data not shown).

**Table 4:** Comparison of medical assistant vs. control group arm on overtime changes on diabetes processes of care and intermediate outcomes of care

	Medical Assistant (MA) Arm (*n* = 644)			MA Control Group Arm (*n* = 2315)			
	Pre	Post	*∆* Over Time	Pre	Post	*∆* Over Time	Difference-in-Difference
Glycated hemoglobin (HbA1c) tested (%, 95 CI)	96.1 (92.3, 99.9)	97.1 (94.1, 100)	1.0 (− 1.1, 3.0)	88.0 (83.4, 92.6)	93.8 (91.2, 96.4)	5.8 (3.3, 8.2)***	−4.8 (− 8.0, − 1.7)**
HbA1c controlled below 8.0% (%, 95 CI)	87.4 (83.7, 91.3)	88.0 (84.2, 91.9)	0.6 (− 3.2, 4.4)	91.9 (90.5, 93.4)	90.8 (89.3, 92.4)	−1.1 (− 2.4, 0.2)	1.7 (− 2.3, 5.7)
Low density lipoprotein cholesterol (LDL-C) tested (%, 95 CI)	96.8 (93.3, 100)	97.2 (94.2, 100)	0.4 (− 1.4, 2.3)	92.4 (89.1, 95.8)	95.0 (92.6, 97.3)	2.5 (1.0, 4.1)**	− 2.2 (− 4.5, 0.0)
LDL-C- controlled below 100 mg/dL (%, 95 CI)	47.6 (39.7, 55.4)	56.0 (47.9, 64.1)	8.4 (0.8, 16.0)*	65.4 (61.6, 69.2)	68.5 (64.8, 72.2)	3.1 (0.2, 6.0)*	5.3 (− 2.8, 13.4)
Blood Pressure- controlled below 140/90 mmHg (%, 95 CI)	65.1 (49.0, 81.2)	69.0 (53.7, 84.3)	3.9 (− 2.0, 9.8)	64.2 (56.6, 71.8)	65.3 (57.8, 72.8)	1.1 (− 1.4, 3.6)	2.8 (− 3.6, 9.2)

Note: Control variables used for adjustment: patient age, sex, and insurance status. Bold denotes statistically significant (*p* < 0.05) differences between the intervention and control group. Statistically significant differences are denoted in the following manner: * *p* < 0.05, ** *p* < 0.01, ***p < 0.001

### Patient-reported measure results

There were no differential changes in the CG-CAHPS Communication and Access to Care composite scores over time across the study arms (Table 5). Patients in the MA intervention arm reported improved chronic care experiences (PACIC-11) (*β* =7.5, *p* < 0.001) and had significantly greater (*p* < 0.05) improvement compared to control group patients.

**Table 5:** Patient experience changes and diabetes outcome changes over time for survey respondents, community health worker vs. medical assistant vs. control group

	Community Health Worker (CHW) Arm (*n* = 166)			Medical Assistant Arm (*n* = 171)			Control Arm (*n* = 361)		
	Pre	Post	*∆* Over Time	Pre	Post	*∆* Over Time	Pre	Post	*∆* Over Time
Patients’ Experiences of Care (Mean, 95% Confidence Interval)									
Communication (CG-CAHPS)	68.6 (60.2, 77.1)	69.1 (60.5, 77.6)	0.5 (− 3.9, 4.9)	74.2 (64.5, 83.9)	76.1 (66.3, 85.9)	1.9 (− 2.4, 6.1)	71.1 (66.2, 75.9)	70.1 (65.2, 74.9)	−1.0 (− 4.0, 2.0)
Access to Care (CG-CAHPS)	40.5 (30.0, 51.0)	41.2 (30.7, 51.8)	0.7 (− 4.6, 6.0)	62.2 (50.1, 74.4)	60.0 (47.7, 72.2)	− 2.2 (− 7.4, 2.9)	55.7 (49.8, 61.6)	55.2 (49.2, 61.2)	0.5 (− 4.0, 3.1)
Patient Assessment of Chronic Illness Care (PACIC-11)	51.7 (46.0, 57.4)	53.8 (48.1, 59.5)	2.1 (− 2.0, 6.2)	47.5 (41.4, 53.6)	55.0 (48.9, 61.2)	7.5 (3.6, 11.5) ***	48.2 (44.8, 51.5)	51.8 (44.8, 51.5)	3.6 (0.7, 6.5)*
Patient Symptoms									
Problem Areas In Diabetes (PAID-5) (higher is better)	50.0 (44.6, 55.4)	51.9 (46.4, 57.3)	1.9 (− 2.7, 6.4)	61.4 (56.0, 66.9)	59.0 (53.4, 64.5)	−2.4 (− 6.8, 2.0)	54.4 (51.1, 57.7)	57.8 (54.5, 61.1)	3.4 (0.2, 6.6)*
Hypoglycemic Events (% reporting an event in past 4 weeks)	51.9 (39.1, 64.7)	51.4 (38.7, 64.2)	−0.5 (− 13.7, 12.9)	46.2 (34.3, 58.2)	52.7 (40.7, 64.8)	6.5 (− 6.3, 19.2)	49.6 (41.5, 57.7)	53.5 (45.4, 61.6)	3.9 (− 5.4, 13.2)

Note: *CG-CAHPS* Clinician Group Consumer Assessment of Health Care Providers and Systems survey; Control variables used for adjustment: patient age, sex, self-rated health, patient-reported use of insulin, and insurance status. Statistically significant over time differences compared to the control groups are denoted in the following manner: * *p* < 0.05; Bold denotes statistically significant (*p* < 0.05) differences between an intervention and control group

Control group patients reported fewer symptoms of diabetes-related emotional distress (PAID-5) over time (*β* =3.4, *p* < 0.05), but the improvement was no different than changes reported by MA or CHW intervention patients. Self-reported hypoglycemic events did not differentially change over time by study arm.

### Key informant interview results

The key informant interviews of control group CHC site directors revealed the positive influence of data collection activities associated with project participation on improved documentation, outreach, and in-reach efforts for diabetes care management. This may be one reason why patients of MA control group sites improved HbA1c testing and CHW control sites improved LDL-C testing during the intervention year.

Even though MAs and CHW roles placed different emphases on panel management vs. health coaching in their training, all new personnel in the intervention sites considered health coaching to be their most important job responsibility. In the intervention clinics, MAs and CHWs performed similar duties as health coaches for patients with diabetes, including providing patient education, goal setting, action planning, and routine monitoring. MAs and CHWs reflected on the valuable training they received to implement the new team approaches: “As we were taught to do in training, forcing is not a good way to achieve the goals. Most of the time, I will let patients make the decision”. The key informants in the control clinics also considered health coaching to be a very important skill, but all acknowledged that their clinics had not implemented any health coaching responsibilities for staff.

Post-implementation interviews revealed that the project personnel were fully integrated into the diabetes care management workflow. Nevertheless, the MA intervention sites were challenged by the fact that MAs did not have enough dedicated time to conduct health coaching activities because regular MA responsibilities often “crowded out” their less time sensitive health coaching responsibilities. The hectic schedule of floor MA duties may interfere with the health coaching, especially on days when demand is high and staffing is low. As one MA indicated, “Sometimes clinic demands interfere- like we need to help out the clinic flow, or when we are short on manpower. Sometimes, a clinic colleague can’t make it that day; then we need to follow doctors to cover for the regular MAs’ jobs”. Two of the three CHW intervention sites used the CHW personnel as health coaches in full-time positions with unique and separated roles from other primary care personnel. This minimized CHWs from being used to cover for missing clinic staff. The CHW health coaches were more satisfied with their dedicated time, which allowed them to build expertise and become specialists in their job without feeling peer pressure, compared to the MA intervention group personnel.

## Discussion

The typical 15-min primary care office visit is widely considered insufficient for meeting the educational and treatment needs of vulnerable patients with diabetes and other chronic conditions. Our study aimed to assess the relative impact of routine implementation of two team-based models that aimed to increase patient contact and support improved diabetes care through health coaching and panel management in CHCs. We hypothesized that teams that included CHWs would more effectively improve intermediate outcomes of diabetes, while MAs would be instrumental in improving processes of care. Diabetes care improved in CHCs integrating CHWs and MAs, but neither team-based approach consistently improved both diabetes process or outcome measures. Our results indicate that the intervention groups modestly improved different intermediate outcomes relative to the control groups, i.e., blood pressure control for CHWs and LDL-C control for MAs.

Our key informant interviews results provide important context for these findings, as new roles and responsibilities were found to be nearly identical for MAs and CHWs, with all sites having a high emphasis on health coaching responsibilities. Given the common emphasis patient care responsibilities for new members across clinics and the common MA backgrounds of most intervention personnel, the intervention effects on improved LDL-C control for patients of the MA arm may be a function of the quality improvement of CHC sites rather than a reflection of generalizable benefits of one model on specific intermediate outcomes of care. Capacity building required of CHC participation may “contaminate” the study by improving control clinic documentation and care processes, but process improvements did not lead to improved intermediate outcomes for patients of control group clinics. To our surprise, there were negative DIDs for Hba1c testing among the MA group and LDL-C testing among the CHW group, which could not be explained by our key informant interviews and observations.

We anticipated small improvements in patients’ experiences of care for the MA and CHW intervention groups. There were no differential changes in patient experiences between intervention groups and the control group, except for better experiences of chronic care among patients in the MA intervention arm. There is some concern that expanded use of team-based models of primary care will erode PCC and patient relationships because of care coordination and team communication challenges associated with shared care arrangements. While patients’ experience scores were generally low and few improvements were observed, it is reassuring that the stressful organizational change of integrating new team roles and responsibilities did not disrupt existing relationships or patients’ experiences of access to care.

Our results should be considered with some important limitations in mind. First, we were unable to control for race/ethnicity in regression model due to collinearity issues. Future cluster randomized trials of CHCs should include racial/ethnic composition as a cluster variable because of the challenge of disentangling site vs. patient compositional effects in cluster randomized intervention studies. Second, we experienced the challenge of unbalanced data in cluster randomized intervention trials of modest sample size. We partially addressed this methodological challenge through matching, weighting, and statistical adjustment, but we were not able to perfectly match patients on all study variables, including BMI, co-morbidities and CHC visits, where small non-significant differences were observed. Third, generalizability is limited due to patient sample attrition and survey non-response. The responses rates to the patient surveys, however, are high for multisite patient experience surveys among CHC patients. Finally, there were substantial pre-intervention differences in the overall quality of diabetes care between the arms based on the practices’ underlying patient characteristics that were not ascertained prior to site randomization. This complicated the direct comparison of MA and CHW intervention arms. Because of this design challenge, we constructed separate matched control groups to enable a more valid estimation of intervention effects. Cluster randomized trials with much larger sets of clinics would reduce the risk of unbalanced allocation of clinics to study arms and could be pursued in the future to minimize these potential biases. Finally, our data were not able to clearly elucidate why specific process and outcome measures improved differentially for the CHW vs. MA team-based models. While our key informant interviews identified barriers, a more robust assessment of fidelity of implementation [40] may have better elucidated what contributed to differential intervention effects for the CHW vs. MA models. Despite these limitations, our study’s pragmatic, mixed methods cluster randomized trial design offers important insights about the benefits and challenges of implementing team-based approaches for diabetes care management in resource limited CHC settings.

In conclusion, while the team-based interventions examined had some effects on improving diabetes care management, competing demands on staff time impeded the routine integration of new roles and responsibilities for managing diabetes care, especially health coaching. Even though CHW and MA team-based models were relatively low-cost personnel, adoption and implementation in routine practice can still be financially challenging for CHCs. Limited reimbursement of non-clinical staff is a major limitation to the dissemination of team-based models that expand the role of these allied health workers to aid patients in diabetes care management. Innovative payment models, such as the Federally Qualified Health Center Alternative Payment Methodology in Oregon [41], which incentives containment of total costs of care, might stimulate the use of team-based diabetes care management models that include CHWs and MAs, as well as peers [42].

## Abbreviations

BMI: Body-mass index
CG-CAHPS: Clinician & Group - Consumer Assessment of Healthcare Providers and System
CHCs: Community health centers
CHWs: Community health workers
DID: Difference-in-difference
HbA1c: glycated hemoglobin
HEDIS: Healthcare Effectiveness Data Information Set
ICD-9: International Classification of Diseases, Ninth Revision
LDL-C: low-density lipoprotein cholesterol
MAs: Medical assistants
PACIC-11: Patient Assessment of Chronic Illness Care
PAID-5: Problem Areas in Diabetes scale
PCCs: Primary care clinicians
SUPREME-DM: Surveillance, Prevention, and Management of Diabetes Mellitus
